# A systematic review of sexual and reproductive health interventions for young people in humanitarian and lower-and-middle-income country settings

**DOI:** 10.1186/s12889-020-08818-y

**Published:** 2020-05-12

**Authors:** Alethea Desrosiers, Theresa Betancourt, Yasmine Kergoat, Chiara Servilli, Lale Say, Loulou Kobeissi

**Affiliations:** 1 Boston College School of Social Work, 140 Commonwealth Avenue, Chestnut Hill, MA 02467 USA; 2 Department of Sexual and Reproductive Health Research, World Health Organization, 20 Avenue Appia, 1211 Geneva 27, Switzerland

**Keywords:** Sexual health, Reproductive health, Young people, Humanitarian settings

## Abstract

**Background:**

Accessibility of sexual and reproductive health (SRH) services in many lower-and-middle-income countries (LMICs) and humanitarian settings remains limited, particularly for young people. Young people facing humanitarian crises are also at higher risk for mental health problems, which can further exacerbate poor SRH outcomes. This review aimed to explore, describe and evaluate SRH interventions for young people in LMIC and humanitarian settings to better understand both SRH and psychosocial components of interventions that demonstrate effectiveness for improving SRH outcomes.

**Methods:**

We conducted a systematic review of studies examining interventions to improve SRH in young people in LMIC and humanitarian settings following Preferred Reporting Items for Systematic Reviews and Meta-analysis (PRISMA) standards for systematic reviews. Peer-reviewed journals and grey literature from January 1, 2000 to December 31, 2018 were included. Two authors performed title, abstract and full-text screening independently. Data was extracted and analyzed using a narrative synthesis approach and the practice-wise clinical coding system.

**Results:**

The search yielded 813 results, of which 55 met inclusion criteria for full-text screening and thematic analysis. Primary SRH outcomes of effective interventions included: contraception and condom use skills, HIV/STI prevention/education, SRH knowledge/education, gender-based violence education and sexual self-efficacy. Common psychosocial intervention components included: assertiveness training, communication skills, and problem-solving.

**Conclusions:**

Findings suggest that several evidence-based SRH interventions may be effective for young people in humanitarian and LMIC settings. Studies that use double blind designs, include fidelity monitoring, and focus on implementation and sustainability are needed to further contribute to this evidence-base.

## Background

Sexual and reproductive health (SRH) and access to sexual and reproductive health services are basic human rights. Based on the sustainable development goals (target 3.7), universal access to sexual and reproductive health services should be attained by 2030. However SRH knowledge and service use remain limited in many lower-and-middle-income countries (LMICs) as well as in fragile settings such as humanitarian emergencies. It is estimated that nearly two billion people worldwide live in areas affected by conflict and violence, and the number of forcibly displaced people is at its highest record to date, at over 68.5 million people worldwide [1]. Of those people in need of humanitarian assistance, an estimated 34 million are adolescent girls and women of reproductive age [2, 3]. LMICs and regions experiencing humanitarian emergencies typically have limited resources and infrastructure to support sexual and reproductive health services, resulting in poor SRH outcomes and limited SRH service utilization [4–6]. The minimal initial services package (MISP) was developed to respond to reproductive health needs at the onset of crisis and includes the following six objectives: identifying an organization to lead MISP implementation, preventing sexual violence and responding to the needs of survivors, preventing the transmission of ad reducing morbidity and mortality due to human immunodeficiency virus (HIV) and other STIs, preventing excess maternal and newborn morbidity and mortality, preventing unintended pregnancies and planning to integrate comprehensive SRH services into primary health care. Despite efforts to improve the availability and uptake of the (MISP), unmet SRH needs remain high and are particularly dire for young people affected by humanitarian emergencies [4]. There is also a substantial lack of research investigating effectiveness and scale-up of interventions focused on improving SRH outcomes among young people in specific cultural contexts [7, 8]. Further research is needed to better understand which SRH interventions have demonstrated effectiveness for improving SRH outcomes in LMIC and humanitarian settings in order to increase evidence-based practices and inform decisions to invest in scaling-up of effective intervention packages.

Poor sexual and reproductive health affects both young men and women, but it is particularly problematic among young women and girls in LMIC and humanitarian settings [9, 10]. Research suggests that humanitarian crises can further compound the risks associated with poor SRH and limited service availability for young women in these contexts [11, 12]. Inadequate SRH service provision has been linked with unintended pregnancies, complications related to unsafe abortions, gender-based violence, and increases in HIV and sexually transmitted infections (STIs) [13]. It is estimated that each year, 12 million adolescent girls and women give birth, and 3.2 million have an unsafe abortion in humanitarian settings [14]. Further, pregnancy during adolescence has been associated with numerous adverse outcomes, including social, economic, and health problems for both young mothers and their children [9, 14]. Access and use of SRH services among young women in LMIC and humanitarian settings is limited, even when services are available [13, 15]. Despite having minimum standards to guide service provision, access to family planning, SRH interventions, antenatal care, and services for sexual violence all remain low [2, 5, 16].

Further, for young people in humanitarian and LMIC settings, comorbid mental health disorders can further compound the risks for poor SRH outcomes and respectively impact SRH service use. Mental health disorders account for approximately 16% of the global burden of disease among young people aged 10–19 [17]. Young people living in humanitarian and fragile settings, including refugees and displaced persons, are at an even higher risk of developing mental health problems due to their living conditions [17, 18]. These risks are further heightened among young women and girls because they are more likely to develop mental health problems (e.g., depression, anxiety, psychological distress) and to experience sexual violence in comparison with men and boys [19, 20]. The high prevalence of mental health problems among young people in humanitarian emergencies is especially concerning because mental health problems have been associated with greater levels of risky sexual behaviors, such as inconsistent contraceptive use, which increases the likelihood of unwanted pregnancies and unsafe abortions as well as the likelihood of contracting HIV and other STIs [21]. Preliminary evidence supports the notion that SRH interventions for people with mental health disorders can reduce sexual risk taking [22]. Integrating psychosocial components that address mental health challenges into SRH intervention packages could therefore enhance overall intervention outcomes; however, the evidence base evaluating the effectiveness of integrated SRH packages in humanitarian emergencies is virtually absent.

Improving SRH outcomes and services among young people in LMIC and humanitarian settings is of critical importance for global public health. With this in mind, this systematic review aims to explore, describe and evaluate more rigorously tested SRH interventions for young people in LMIC and humanitarian settings to better inform the evidence-base on interventions that demonstrate effectiveness for improving SRH outcomes. A stronger evidence base is needed to better understand what types of SRH interventions work for which populations of young people in a given context, as well as what components of effective interventions might be common across those different interventions. This review also explored the psychosocial components of interventions to better understand common psychosocial practice elements of SRH interventions.

Given the high rates of mental health problems among young people in humanitarian and fragile settings and the strong link between mental health problems and sexual risk behaviors, incorporating trauma-informed psychosocial components into SRH interventions could improve SRH outcomes, particularly those related to service use. Findings from this review could help inform the development of integrated health promotion and prevention policies and programs to address the significant combined burden of poor SRH and mental health outcomes for vulnerable populations of young people in LMIC and humanitarian settings.

## Methods

This systematic reviewed followed the Preferred Reporting Items for Systematic Reviews and Meta-Analysis (PRISMA) statement and standards for systematic reviews [23]. This review was registered in the PROSPERO database (Prospero # CRD42019123233).

### Search strategy and selection criteria

SRH search terms were selected based on the standard definition from the International Conference on Population Development (1994) and the World Health Organization’s (WHO) SRH strategies and guidance (2010). Search terms for SRH were generated by the authors in consultation with a WHO librarian and encompassed the following: general sexual and reproductive health, pregnancy, family planning, contraception, abortion, prenatal healthcare, antenatal health care, HIV/AIDS, STIs, prevention of mother-to-child transmission (PMTCT), maternal and newborn health, gender-based violence, and adolescent sexual health. We included studies conducted in the Middle East, low-income, lower-middle income, and middle-income countries as defined by the current World Bank country classifications using individual country names in the search. In addition to general humanitarian-crisis related terms, the WHO designations for current humanitarian emergencies (at levels 1, 2, and 3) were also used to guide identification of humanitarian settings. These included any type of humanitarian crises whether, man-made or natural disasters. We focused on LMICs because the resources available to address humanitarian crises in these settings are much different than those available in high-income countries, and a significant proportion of displaced persons seeking refuge from humanitarian emergencies are hosted by developing countries [2].

Our search strategy used the following format: (SRH related terms) and (intervention or education related terms) and (country/setting related terms). We restricted the search to randomized controlled trials, adolescent and young adult (ages 13–29) populations, and publications in the English language. We included studies only in peer-reviewed journals published between 2000 and 2018 and searched the following four databases: Pubmed, Psycinfo, Medline, and Embase. We then conducted a second search of upper-middle income countries using the same search strategy and hand-selected studies for title/abstract screening that were randomized controlled trials of SRH interventions among adolescents and/or young adults ages 13–29. We conducted this additional search in upper-middle income countries in order to more thoroughly review the literature on effective SRH interventions for young people. We also searched the grey literature by targeting international organizations involved in humanitarian work and reviewing any listed publications that included our SRH search terms. Finally, reference lists of prior systematic reviews on SRH domains in humanitarian settings were reviewed for potentially relevant studies. These multiple complementary search strategies helped ensure that we were as exhaustive as possible in our search strategy.

### Inclusion/exclusion criteria

For studies in LMICs, we included only randomized controlled trials among adolescents and/or young adults ages 13–29. For Middle Eastern countries and humanitarian settings, we loosened our inclusion/exclusion criteria such that pilot randomized controlled trials and quasi-experimental designs among participants ages 10–49 (those of reproductive age) were considered for inclusion because we anticipated that there might be few RCTs on SRH interventions among adolescents and young adults in these settings. We also included studies that focused only on mental health outcomes if they met all of our search criteria because identifying effective interventions for improving mental health in these settings could help identify effective approaches for addressing mental health concerns among those who also have unmet SHR needs as well as inform the development of psychosocial components of SRH intervention packages in these contexts. Relevant interventions that targeted our specified outcomes included: educational, psychosocial, prevention, community-based, psychoeducational, empowerment, mental health, psychological, counseling, family-based, and training programs.

Exclusion criteria were as follows: studies conducted in high-income countries, secondary analysis of primary data, and evaluations of non-relevant interventions (medical, pharmacological, dietary, exercise or cash-transfer interventions).

### Data analysis

All citations returned from the search databases and were downloaded and entered into a Microsoft excel database for screening of titles and abstracts based on our inclusion criteria. Data was then extracted from those studies selected for inclusion into another Microsoft excel database and subject to a secondary full text screening.

Extracted data included: first author, year of publication, title, trial duration, participant characteristics (age, gender), setting characteristics, country in which study took place, study design, type of intervention, intervention components, session topics (if available), recruitment incentives, follow-up incentives, intervention length, session format, follow-up duration, control condition, sexual and reproductive health outcomes, mental health outcomes and key findings.

**Fig. 1:**
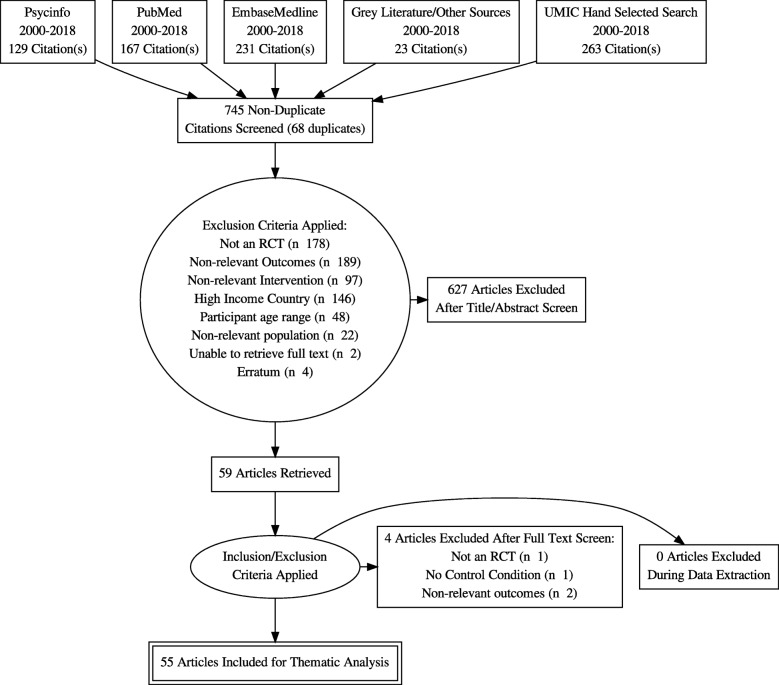
PRISMA Flow Chart

For primary screening of titles and abstracts and secondary full text screening, two authors independently conducted screening and then cross-checked for consistency. Discrepancies were resolved via discussion between the two authors. If no agreement could be made, a designated third reviewer was consulted (see Fig. 1).

We used principles derived from a narrative approach for data synthesis and conducted a thematic analysis of included studies due to the diverse range of study outcomes and intervention approaches. The narrative synthesis approach is an iterative process that involves developing a theory about how and why the intervention works and developing a synthesis of findings for included studies [24]. Findings were analyzed by SRH outcomes, intervention components (SRH and psychosocial components), intervention delivery information, and key findings (see Table 1). Themes for SRH intervention components were developed iteratively after manually coding the papers and extracting relevant data. Coding for SRH intervention components was conducted by two coders and cross-checked for consistency. Psychosocial intervention components were coded using the Practice Wise Clinical Coding system [80], which is an empirically derived and tested system for identifying common practice elements across different interventions. There were four discrepancies in coding of psychosocial components. Discrepancies in coding were resolved by discussion until consensus was obtained. Intervention components that were not captured by the existing codes were recorded as free text and reviewed to generate additional practice codes, which were cross-checked by the second coder. We identified seven additional intervention practice codes: (a) emotion regulation, (b) resilience building, (c) coping skills, (d) creative expression, (e) self-efficacy, (f) behavioral activation, and (g) interpersonal skills.

**Table 1:** Thematic Analysis of Articles Included Following Full Text Screening

Author/Year	Country/Setting	Population	Psychosocial Components	SRH components	Study Design	Delivery Information	Key Findings
Humanitarian Crisis Settings (Defined by WHO) and Refugee Populations							
Cowan et al. 2010 [25]	Zimbabwe	Girls and boys ages 18–22	Cognitive	Youth: HIV prevention, sexual risk behavior, gender equity; Community: adult support of youth SRH, safe spaces	Cluster RCT: 2 arm	School-based youth groups (4 years, 24 sessions in year 4); Parent + community stakeholder program (22 sessions); Nurse training program in clinics	Intervention: sig increase in STI knowledge, & pregnancy prevention; no effects on HIV knowledge, sexual behavior, clinic attendance or HIV rates; increase in condom negotiation self-efficacy for females only at 4 yrs
Stark et al. 2018 [26]	Ethiopia (refugees)	Girls and boys ages 13–19	none	Creating safe spaces, economic empowerment	Quasi-Experimental	Community-based groups; 10 90 min weekly sessions; 10 caregiver discussion groups	No sig intervention effects on SRH outcomes
USAID 2017 [27]	Ethiopia	Girls and boys ages 10–24	none	YFS: Family planning service delivery for youth; contraception education & provision	Quasi-Experimental: 2 arm	Counseling & provision of contraception at clinics; Family planning outreach activities	Rates of new acceptors of contraception higher at intervention sites post-intervention, but no statistical differences; uptake of implants increased at interventions sites; no difference for IUDs
Ezeanolue et al. 2016 [28]	Nigeria	Male partners, avg. age = 38	none	Pregnancy education, ANC care education, HIV transmission, HIV integrated services	Cluster RCT: 2 arm	Church-based/family-based; 1 session: pregnancy and health education via a “game show”	Male partners in intervention group showed higher rates of HIV testing compared with controls at post-intervention (post-delivery)
Ishola et al. 2015 [29]	Nigeria	HIV+ pregnant women	Mindfulness, Cognitive, Goal Setting	HIV post-test counseling	Solomon 4-group	PMTCT Center groups; 1 ACT session + weekly text messages for 3-months	Intervention: sig improvements in psychological flexibility at post-intervention
Okonofua et al. 2003 [30]	Nigeria	Girls and boys ages 14–20 (*n* = 1896)	none	Reproductive health education, STI prevention & referrals; clubs & campaigns in school, public lectures	RCT: 2 arm	School-based RH clubs; Peer-educator training, peer delivered counseling (individual or group-based), peer outreach; Health provider training in STI treatment (30 h of lectures)	Intervention: sig higher STI knowledge, awareness of partner’s STIs, & STI treatment-seeking; sig reduced STI prevalence at post-intervention
Mercy Corps 2015 [31]	Niger	Girls ages 10–18 (*n* = 829)	Support networking (Safe spaces: SS)	RH education, risk of early pregnancy (Livelihood Training)	Post test with a control	Community-based groups; 87–91 h of direct intervention (SS over 9 months, SS+ Livelihood Training over 19 months)	Sig reductions in attitudes on contraceptive use & age to have a baby in both groups at post-intervention; SS only had sig higher RH knowledge
Bass et al. 2013 [32]	Dem Rep Congo (DRC)	Women avg. age = 36.9; 33.8	Cognitive	none	Mixed Method	Community-based groups; 1 individual session + 11,120 min group sessions	Intervention: sig improved anxiety, PTSD symptoms, depression
OCallaghan et al. 2013 [33]	Dem Rep Congo	Girls ages 12–17 (war affected)	Relaxation, problem solving, coping skills, emotion regulation, cognitive, psychoeducation	none	RCT: 2 arm	15,120 min sessions 3 days per week	Intervention: sig reduced PTSS symptoms, distress, anxiety, depression at post-intervention
Panter-Brick et al. 2018 [34]	Jordan (Syrian Refugees)	Girls and boys ages 12–18	Support networking, communication skills, relaxation, psychoeducation, resilience building	Gender equity, creating safe spaces	RCT: 2-arm	Community youth center groups; 2 sessions per week for 8 weeks; Structured group activities	Intervention: sig improved MH, insecurity & distress at post-intervention; sustained effects for distress at 1-yr follow-up
Langhe-Nielson et al. 2011 [35]	Palestine (Gaza) Refugees	Girls and boys ages 12–17	Narrative	none	Quasi Experimental	School-based groups in camps; 2 15 min writing sessions on traumatic memories for 3 days	No significant effects of intervention
Barron et al. 2016 [36]	Palestine	Girls and Boys ages 11–15	Coping skills, relaxation psychoeducation, exposure	none	RCT: 2 arm	School-based groups; 5 sessions: Teaching Recovery Techniques (TRT)	Intervention: sig reduced PTSD symptoms at post-intervention
Punamaki et al. 2014 [37]	Palestine (Gaza)	Girls and boys ages 10–13	Coping skills, relaxation psychoeducation, exposure	none	RCT	School-based groups after school; 2 sessions per week for 6 weeks: Teaching Recovery Techniques (TRT)	No sig differences in emotion regulation (ER) at post-intervention, ER intensity mediated intervention effects on MH outcomes
Qouta et al. 2012 [38]	Palestine (Gaza)	Girls and boys ages 10–13	Coping skills, psychoeducation, creative expression	none	Cluster RCT: 2-arm	School-based groups; 2 sessions per week for 4 weeks; 1 psychologist per group	Intervention: sig reduced clinical PTSS in boys only compared with controls at post-intervention
Diab et al. 2014 [39]	Palestine	Girls and boys ages 10–13	Relaxation, emotion regulation, problem solving, psychoeducation	none	Quasi Experimental	School-based groups (TRT); Delivered by trained counselors; Structured group activities; Homework to practice relaxation	No sig effects of intervention on MH outcomes at post-intervention
Kalantari et al. 2010 [40]	Iran (Afghani refugees)	Girls and boys ages 12–18	Narrative	none	Pre-post	School-based groups; 2 15 min sessions per day on trauma memories for 3 days	Intervention: sig decrease in traumatic grief at post-intervention (small sample size; n ~ 30)
Mon et al. 2017 [41]	Myanmar (HIV+ parent)	Girls and boys ages 10–16	Mindfulness, relaxation,	RH education: (puberty, HIV/STIs contraception, pregnancy)	RCT: 2- arm	Community center groups; 3 monthly sessions conducted by 3 trained instructors; Homework to practice meditation	Intervention: sig higher RH knowledge at 3 mos but not 6 mos; sig higher emotion regulation & interpersonal effectiveness
Mon et al. 2016 [42]	Myanmar (HIV+ parent)	Girls and boys ages 10–16	Mindfulness, relaxation,	RH education	Cluster RCT: 2- arm	Community center groups; 3 monthly sessions; Homework to practice meditation	Intervention: sig lower conduct and emotional problems at 6 mos follow-up
Newmann et al. 2016 [43]	Kenya	HIV+ men and women ages 18–45	none	Family planning talks, provision of condoms & effective contraception	RCT: 2 arm	Family planning integrated into HIV services	Intervention site: sig higher gender equity attitudes in men only, sig more effective contraception use in women only at 1 yr follow-up
Bryant et al. 2017 [44]	Kenya	Women, avg. age = 35	Behavioral activation, problem solving, relaxation, support networking	none	RCT: 2 arm	Home-based individual sessions; 5 weekly 90 min sessions (Problem Management: PM+)	Intervention: sig greater reductions in distress; reductions in functional impairment; no differences in gender based violence at 6 mos follow-up
Dawson et al. 2016 [45]	Kenya	Women, avg. age = 33 affected by GBV	Behavioral activation, problem solving, relaxation, support networking	none	Pilot RCT: 2 arm	Home-based individual sessions; 5 weekly 90 min sessions (PM+)	Intervention: sig reductions in PTSD symptoms at post-intervention; no sig differences in distress or functional impairment
Baiocchi et al. 2017 [46]	Kenya	Girls and boys ages 10–16	Girls Education: assertiveness training, problem solving, emotion regulation	Boys education: gender equality (Gender equity), sexual assault prevention (GBV),	Cluster RCT: Matched pairs	School-based groups (boys and girls separate); 6 weekly 120 min sessions + 1 booster session at 3-mos; Structured group activities	Intervention: sig increase in self-efficacy (perceived ability to cope with stress) and decrease in “estimated” rate of sexual assault at post-intervention
Puffer et al. 2016 [47]	Kenya	Girls and boys ages 10–16	Modeling, problem solving, goal setting, coping skills, communication skills	HIV education & prevention, economic empowerment	Cluster RCT: 2 arm	Family-based/church-based; 9120 min sessions; Parent groups, youth groups (boys and girls separated), & church leader discussion groups	Intervention: sig improved family communication at 1 & 3 mos, higher self-efficacy for safe sex at 1 mo; no effects on beliefs about sexual risk; marginal effects for HIV knowledge
Cohen et al. 2017 [48]	Kenya	HIV+ Women ages 18–45	none	Integration of HIV & FP services; family planning counseling	Cluster RCT: 2 arm	Family counseling provided at HIV clinics	Sig increase in use of effective contraception, decrease in pregnancy rates at 1 & 2 yrs.
Turan et al. 2015 [49]	Kenya	HIV+ mothers and infants	none	Integration of PMTCT and HIV care with antenatal care services	Cluster RCT: 2 arm	“Week-long” health care provider training on HIV, PMTCT and ANC care & service promotion	Intervention: sig higher HIV care enrollment at 1 yr, more likely to initiate & use ART during pregnancy
Adam et al. 2013 [50]	Kenya	1st & 2nd yr University Students	none	HIV prevention: condom use, monogamy, abstinence	RCT: 2 arm	University-based/peer-delivered; Peer educator training; 32 h over 4 weeks	No sig differences between groups at post-intervention
Grossman et al. 2011 [51]	Kenya	HIV+ women ages 18–45	none	Family planning services integrated into HIV clinics	Cluster RCT: 2 arm	Trained peer educators delivered family planning education in groups (sessions not described)	Intervention: sig higher odds of using effective contraception at 1 yr, no sig difference in condom use
Lower Income Country Settings							
Penfold et al. 2014 [52]	Tanzania	Women ages 13–49 and infants	none	Antenatal care education	Cluster RCT: 2 arm	Home-based individual sessions; 3 sessions pre-birth + 1 post-birth; Delivered by trained volunteers	Sig higher reports of delaying first birth, exclusive breastfeeding, and cord cutting hygiene post-delivery
Magoma et al. 2013 [53]	Tanzania	Pregnant women avg. age = 25	none	Birth planning education integrated into ANC services	Cluster RCT: 2 arm	Clinic-based sessions; ANC providers received 2 days of didactic training	Intervention: effects moderated by SES; women more likely to deliver in health unit but not statistically significant for all women
Ross et al. 2007 [54]	Tanzania	Girls and boys avg. age = 15.7	none	Increase provision of youth friendly SRH services, sexual health education, condom promotion/provision	Community RCT: 2 arm	School-based groups; 12 40-min sessions over 1 year; Teacher led & peer-assisted; Community-wide activities (e.g., condom promotion by youth)	Intervention: sig impact on SRH knowledge, HIV/STI knowledge, sexual behavior attitudes at 3 yrs.; no sig effects on HIV or STI prevalence
Jordans et al. 2010 [55]	Nepal	Girls and boys ages 11–14	Creative expression, exposure, narrative, psychoeducation, resilience building	none	Cluster RCT: 2 arm	School-based groups; 15 60 min sessions over 5 weeks; Structured groups activities	Intervention: moderate reductions in psychological problems for boys, increased prosocial behavior for girls at post-intervention
Ssewamala et al. 2010 [56]	Uganda (Aids Orphaned)	Girls and boys avg. age = 13.7	none	Economic empowerment	Quasi RCT	School-based groups; 12 sessions on financial planning; monthly peer mentorship meetings for 10 months	Intervention: sig improved sexual risk taking attitudes for boys at post-intervention; girls showed increased approval of sexual risk-taking
Bolton et al. 2007 [57]	Uganda	Girls and boys ages14–17	Interpersonal therapy; creative expression	none	RCT: 2 arm	Groups held in displaced person camps; 16 weekly 90–120 min sessions	Intervention: girls only showed sig improvements in depression at post-intervention; no effect on anxiety
Devries et al. 2017 [58]	Uganda	Girls and boys ages 11–14	Goal setting, psychoeducation- staff	Sexual/emotional violence education; power in relationships	Cluster RCT: 2 arm	School-based group sessions; Good Schools Toolkit: activities for students and staff	Intervention: sig reduced levels of violence (including sexual) at 3 mos follow-up
Atwood et al. 2012 [59]	Liberia	6th grade girls and boys (*n* = 812)		HIV prevention, condom use attitudes, perceived sexual risk, sexual refusal self-efficacy; condom negotiation self-efficacy	Match Group RCT: 2 arm	School-based groups; 1 male & 1 female health educator delivered education in health class weekly over 8 weeks	Intervention: sig improved attitudes about condoms, increased condom use at 9 mos; no effect on age first sex or multiple sex partners
Hossain et al. 2013 [60]	Cote d’lvoire	Men avg. age = 32	none	Gender-based violence, healthy relationships	Pilot RCT: 2 arm	Community program + men’s discussion group; 16 sessions over 4 months	Intervention: sig lower reports of GBV, improved attitudes about GBV at 1 yr follow-up
Gupta et al. 2013 [61]	Cote d’lvoire	Women avg. age = 37	Communication skills	Gender norms & attitudes, economic empowerment, GBV	RCT 2 arm	Community-based groups for women and their male partners; 8 1.5–2.5 h sessions over 16 weeks; Delivered by 1 male & 1 female facilitator	Intervention: acceptance of wife beating reduced at 3 mos, no sig differences in reported IPV or attitudes about sex refusal
Middle Income Settings							
Villaruel et al. 2010 [62]	Mexico	Girls avg. age = 17.6 (*n* = 829)	none	Sexual risk reduction, pregnancy education, contraception, parent-adolescent sex talks	RCT: 2 arm	School-based groups; 6 h of sessions total; Structured group activities; Parent groups	Intervention: sig more likely to be older & use a condom at first sex at 48 mos; no effect on consistent condom use
Kaljee et al. 2005 [63]	Vietnam	Boys and girls ages 15–20 (*n* = 480)		HIV/AIDS Knowledge, effective contraception, intentions to use condoms, sexual decision making skills	RCT	School-based groups; 10 weekly 2 h sessions; 1 facilitator per group (same gender groups); Parent groups	Intervention: sig greater HIV/AIDS knowledge; condom use self-efficacy and condom negotiation self-efficacy; perceived efficacy of condoms; intentions to use condoms at post-intervention and 6 mos
Leventhal et al. 2016 [64]	India	Girls, avg. age = 13	Emotion regulation, assertiveness training, communication skills, problem solving, resilience building	Gender equity, gender based violence; RH heath education (health curriculum)	RCT: 3- arm	School-based groups; 1 session weekly for 21–23 weeks; 2 trained facilitators per group; Structured group activities	Psychosocial curriculum + health curriculum group had sig higher gender equality attitudes and RH health knowledge than controls at post-intervention
Leventhal et al. 2015 [65]	India	Middle school girls	Resilience building, emotion regulation, assertiveness training problem solving, goal setting, communication skills	none	Stratified Block RCT: 4 groups	School-based groups; 23 60 min weekly sessions; Structured group activities	Intervention: sig higher emotional resilience, self-efficacy (belief that one can cope with adversity and perform difficult tasks), well-being at post-intervention; no effect for depression
Raj et al. 2016 [66]	India	Couples; husbands ages 18–30		Gender equity, family planning counseling, contraception education, sexual-risk behaviors	Cluster RCT: 2 arm	Clinic-based or home sessions; 2 individual sessions delivered by male health providers to men & 1 couples session over 3 months	Intervention: women sig more likely to communicate about contraception & use effective contraception at 9 mos, less likely to report IPV at 18 mos; men sig less likely to report acceptance of IPV at 9 & 18 mos; no effect on pregnancy rates
Jewkes et al. 2008 [67]	South Africa	Girls and boys ages 15–26	Communication skills; coping skills	HIV prevention, STIs pregnancy prevention, sexual risk taking, condom use, GBV	Cluster RCT: 2 arm	Community-based groups; 13 3 h sessions (girls and boys separated), 3 peer group meetings, 1 community meeting	Intervention: reduced reported GBV in boys but not significant at p < .05; no sig effects for girls at 2 yrs.; no sig effects on HIV prevalence
Taylor et al. 2014 [68]	South Africa	Girls and boys avg. age = 14 (*n* = 816)	none	Pregnancy prevention, gender norms, education on puberty, decision-making, healthy relationships	RCT: 2 arm	School-based groups; 12 weekly sessions; Structured group activities	Intervention: sig healthier attitudes, intentions for abstinence, plans to communicate with partner about pregnancy, higher reported condom use at 8 mos follow-up
Matthews et al. 2016 [69]	South Africa	Girls and boys avg. age = 13	Assertiveness training, communication skills	HIV prevention, IPV prevention, gender equity, GBV, sexual decision-making, healthy relationships	Cluster RCT: 2 arm	School-based groups after school; 21 60–90 min education sessions; School IPV prevention program; School-based youth friendly health service	No sig differences in sexual risk behavior at 12 mos; intervention sig less likely to report experiencing sexual violence; higher HIV knowledge & condom knowledge
Jones et al. 2013 [70]	South Africa	Pregnant women avg. age = 28	Communication skills, problem-solving, assertiveness training	HIV/STI prevention, contraceptive use, PMTCT service usage	RCT: 2 arm	Clinic-based/Couples-based; 4 weekly 90–120 min couples sessions	Intervention: sig decreased partner violence, increased HIV knowledge, condom use, use of sexual negotiation skills at post-intervention
Mott MacDonald Team 2017 [71]	Zambia	Girls ages 10–14; 15–19	Resilience building	Vouchers for SRH services, economic empowerment, health education	RCT: 2 arm	Weekly meetings over 2 years	No sig evidence for effects on SRH outcomes at 2 yrs
Vance et al. 2013 [72]	Ghana & Zambia	Women postpartum avg. age = 24	none	Family planning messages integrated with immunization services, family planning referrals, LAM education	Cluster RCT: 2 arm	Clinic-based individual sessions; 4 sessions, 30 s each; Vaccinators trained via manual to provide LAM education, family planning messages & referrals	No sig differences between groups and very few women knew LAM criteria at post-intervention
Rockiki et al. 2017 [73]	Ghana	Girls ages 14–24	none	Pregnancy prevention, reproductive anatomy, STI prevention and education, effective contraception education	Cluster RCT: 3-arm	1 text message per week for 12 weeks (unidirectional = RH information; interactive = quiz + feedback and encouragement)	Interactive: sig higher RH knowledge than unidirectional & control at 3-mos; no sig differences at 15-mos
Aninanya et al. 2015 [74]	Ghana	Girls and boys ages 10–19	none	SRH education, sexual attitudes and behaviors; promoting youth SRH, ANC, prenatal, and HIV/STI service usage	Cluster RCT	School-based youth groups; Peer outreach activities; Youth friendly health services provider training (1 session); Community mobilization meetings (50+ sessions); Delivered by government workers	Intervention: sig higher odds of STI, ANC, and prenatal service usage at 3 yrs.; no sig differences in HIV or SRH service usage
Carlson et al. 2013 [75]	Mongolia	Female sex workers, avg. age = 25	Motivation enhancement	HIV risk reduction, gender-based violence	Cluster RCT: 3 arm	Groups held at NGO building; 4 weekly 90 min sessions; 2 wrap-up sessions for MI group	Intervention: sig reductions in violence from paying sex partners in MI alone, MI+ risk reduction, and also controls at 6 mos follow-up
Khan et al. 2017 [76]	Pakistan	Pregnant women ages 18–30	Psychoeducation, relaxation, support networking	none	Pilot RCT: 2 arm	Home-based/family-based; 1 20 min session + 1 60 min session	No sig differences between groups at post-intervention
Bhutta et al. 2011 [77]	Pakistan		none	Antenatal care promotion	Cluster RCT: 2 arm	Community-based groups; quarterly sessions delivered by lay workers	No sig differences between groups
Middle East Non-humanitarian Crisis Settings							
Bastani et al. 2006 [78]	Iran	Pregnant women ages 18–30	Relaxation, psychoeducation	none	RCT: 2 arm	Clinic-based groups; 7 weekly 90 min sessions; Delivered by trained nurses	Intervention: sig lower anxiety & stress post-delivery, sig reductions in low birth weight and C-sections
Berger et al. 2014 [79]	Israel	Girls and boys ages 11–13	Emotional regulation, relaxation	none	Quasi RCT: 2 arm	16 90 min weekly sessions	Intervention: sig reductions in PTSD symptoms, anxiety, somatic symptoms at post-intervention

Note. *Pregnancy prevention was coded as SRH education. **Sig is used as an abbreviation for “significant” and “significantly”. Studies reporting significant effects on at least one SRH outcome are highlighted in grey

**Fig. 2:**
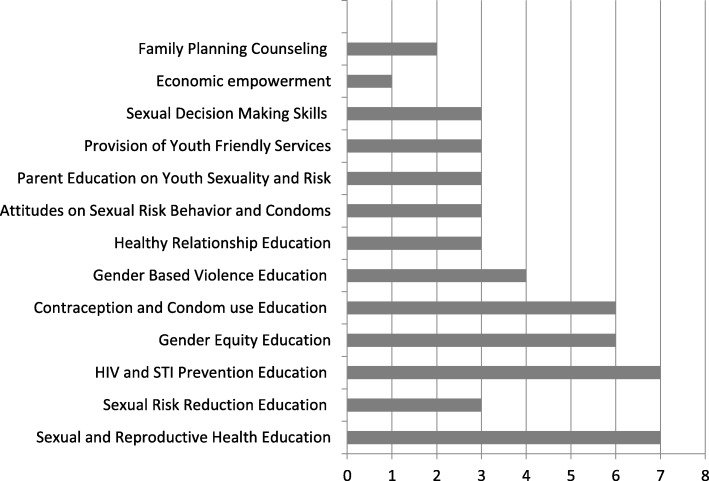
Frequency of Sexual and Reproductive Health Intervention Components for Interventions Demonstrating Significant Effects (n = 17)

**Fig. 3:**
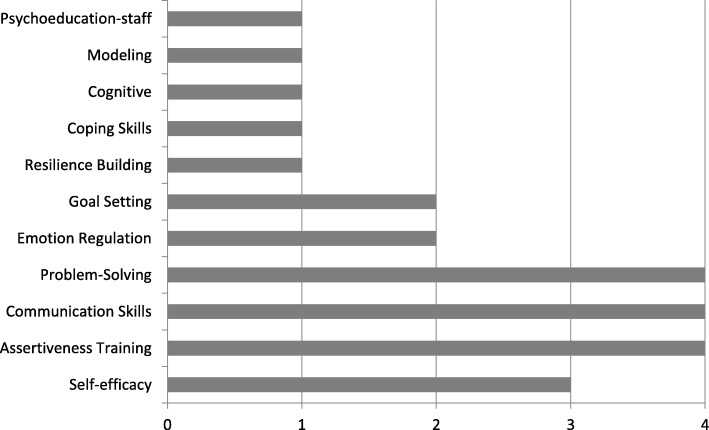
Frequency of Psychosocial Intervention Components for InterventionsDemonstrating Signficant Effects on SRH Outcomes (*n* = 17)

For those studies that reported significant effects of the intervention on SRH or mental health outcomes, summary charts illustrating common SRH and psychosocial components across studies were generated (see Figs. 2 and 3). Interventions were classified as effective by a significant between group intervention effect (*p* < .05) in which the intervention group demonstrated significant improvements in one or more SRH outcomes at the final follow-up assessment in comparison to the control group or to another intervention group. For studies reporting moderator effects where significant effects on given SRH outcomes were reported only for specific sub-groups, these effects are described in Table 1. In cases where moderation was reported, those SRH interventions that reported overall treatment effects or significant effects by gender were considered effective interventions; other moderation effects were not sufficient for an intervention to be considered effective [81]. In our presentation of findings, we focused primarily on those studies demonstrating effectiveness for SRH outcomes and not those that focused exclusively on mental health intervention components and outcomes because a systematic review focusing exclusively on mental health and psychosocial interventions for improving SRH among youth in LMIC and humanitarian settings was conducted previously (Turner et al., 2018; Prospero #: CRD42018081410).

In order to further extract data on common intervention components for those interventions designated as effective, written requests were sent to the authors of papers describing effective SRH interventions to obtain intervention manuals and protocols. Follow-up requests were sent to authors who did not respond (*n* = 7). An online literature search for intervention manual materials was also conducted when authors failed to respond to follow-up requests. Common SRH and psychosocial components were identified through extracting data from intervention manuals, when available, or through the description of intervention components provided in the text of the journal article in cases where treatment manuals were not available (*n* = 8). Two independent raters reviewed intervention manuals and journal articles to extract core psychosocial intervention components. In cases where there was discrepancy (*n* = 2), a third rater reviewed intervention material and consensus was arrived at through discussion.

Methodological reporting quality of included studies was assessed using the Effective Public Health Practice Project (EPPHP) Quality Assessment Tool for Quantitative Studies [82]. Several modifications similar to those described by Brown et al., 2016 were made to better characterize aspects of methodological quality in light of the limited information provided in some studies. Two raters assessed methodological questions independently, and discrepancies were resolved through discussion.

## Results

Results of the search strategy are summarized in the Prisma Flow chart (see Fig. 1). The literature search, review of grey literature, and reference list review yielded 813 results, 745 of which were non-duplicate citations. After title and abstract screening of the 745 studies, 686 were excluded. Full text screening was performed for 59 studies. Four studies were excluded following full text screening, yielding 55 studies for inclusion in thematic analysis. Table 1 presents a summary of the thematic analysis for included studies. 38 out of 55 studies evaluated SRH outcomes, and 17 of these studies reported significant intervention effects on one or more SRH outcomes in our primary population of interest (ages 13–29). For the purposes of consolidating key findings of this review, we discuss below the results from thematic analysis of these 17 studies that reported intervention effectiveness on SRH outcomes over time among adolescents and young adults. Additionally, studies that reported significant effects for populations of adults over age 29 are presented in the thematic analysis table but not discussed in the summary of results because our primary focus was on effective interventions for SRH in adolescent and young adult populations. SRH outcome categories were centered on: (a) effective contraception and condom use skills, (b) HIV/STI prevention and education, (c) sexual self-efficacy, (d) SRH knowledge and education, (e) gender-based violence education. The majority of studies assessed multiple SRH outcomes and some incorporated psychosocial intervention components (see Table 1).

### Effective contraception and condom use skills

We identified eight studies that reported improvement in outcomes related to effective contraception or condom use, intentions to use contraception or condoms, or attitudes about contraception or condom use. All studies were randomized controlled trials except for the Mercy Corps (2015) report [31], which used a “post-test with control” design. Villaruel et al. (2010) evaluated the effects of a school-based sexual risk reduction intervention, “the Cuidate Program”, on youth in Mexico and found that those who received the intervention were significantly more likely to use a condom (OR, 1.75; 95% CI, 1.14–2.69; *P* < 0.05) or other contraception (OR, 1.53; 95% CI, 1.00–2.33; P < 0.05) during their first sexual encounter than those in the control group at 48-month follow-up [62]. However no effect was found for consistent condom use over time. Kaljee et al. (2005) examined the effects of an HIV/STI prevention intervention delivered in schools among Vietnamese youth and found that those who received the intervention reported significantly greater intentions to use condoms (*p* < .05) and perceived efficacy in condom use skills (p < .05) compared with those in the control condition at post-intervention and 6-month follow-up [63]. The authors were unable to measure condom use behavior due to low reported sexual intercourse. In a study of integrated family planning counseling services into HIV clinics among young women in Kenya, those who received integrated services reported significant increases in use of effective contraception (from 31.7 to 44.2% of encounters) at 24-month follow-up compared with controls and decreases in pregnancy rates at 48-month follow-up compared with controls at year one (rate ratio: 0.72; 95% CI 0.60–0.87) [48]. Atwood et al. (2012), examined an adapted version of “Making Proud Choices”, an HIV/STI and pregnancy prevention curriculum, delivered in schools for 6th grade youth in Liberia and found that youth who received the intervention reported significant improvements in positive attitudes towards condoms at 3-month and 6-month follow-up (β = .12, *p* < .05; β = .08, p < .05) [59]. Self-reported condom use self-efficacy was also significantly higher among those who received the intervention at 3-months and 9-months follow-up (β = .08, p < .05; β = .07, *p* < .10) compared with those in the control condition. Among youth who were sexually active, there was a significant effect on frequency of consistent condom use among those who received the intervention compared with controls at 9-month follow-up (β = .34, p < .05). Taylor et al. (2014) investigated a pregnancy prevention intervention delivered in schools for adolescents in South Africa and found that those who received the intervention reported significantly higher condom use than those in the control (54.2% vs. 36.7%, *p* < .01) [68].

Mercy Corps (2015) evaluated a Safe Spaces program compared with Safe Spaces in combination with a livelihood education program and a control condition delivered in schools and homes for girls (ages 10–18) in Niger [31]. The Safe Spaces program incorporates education on life skills, risk associated with early marriage and pregnancy, and reproductive health knowledge, whereas livelihood education focuses on savings and loan information, livestock management, and gardening. The authors reported significant improvements in attitudes towards contraception use in both those who received Safe Spaces (p < .01) and those who received Safe Spaces plus Livelihood (*p* < .05) compared with controls. Matthews et al. (2016) evaluated a school-based HIV and intimate partner violence (IPV) prevention program among youth in South Africa and found that youth who received the intervention reported significantly greater condom knowledge than youth in the control condition at 12-month follow-up (*p* < .001) [69]. The program involved an educational program during school delivered by study research staff, a youth friendly health service in school, and a peer-outreach component to spread information on violence prevention in school. Raj et al., (2016) evaluated a couples-based gender equity and family planning intervention among young married couples in India and found that women in the intervention group were significantly more likely to use effective contraception at 18-month follow-up (AOR = 1.57–1.58, *p* = 0.05) [66]. In a couples-based HIV risk-reduction and PMTCT intervention delivered to young couples in ANC clinics in South Africa, participants in the intervention group had greater odds of increased condom use at post-intervention compared with those in the control group (OR = 5.1, 95% CI (OR) = (2.0, 13.3) [70].

### HIV/STI prevention and education

We identified six studies that reported significant improvements in HIV/STI knowledge, reductions in self-reported STI symptoms or increases in STI service usage. In a study investigating a multi-pronged HIV prevention intervention that included a youth, parent, and community component in Zimbabwe, youth (ages 18–22) who received the intervention reported significantly higher STI knowledge compared (AOR = 1.32; 95% CI: 1.08–1.61) and pregnancy prevention (AOR = 1.59; 95% CI:1.27–1.99), but no differences were found on HIV knowledge [25]. Aninyana et al. (2015) evaluated an SRH education intervention that involved a school-based curriculum for youth, peer-outreach activities, and youth friendly services provider training and found that youth who received the intervention demonstrated higher odds of STI service usage (AOR 2.47; 95%CI: 1.78–3.42), but no significant differences on HIV testing service usage [74]. Another study that investigated an intervention involving school-based educational sessions delivered by teachers, community activities (i.e., community-based condom promotion by youth), and provision of youth friendly services found that youth who received the intervention reported significantly greater HIV transmission knowledge (male rate ratio: 1.44 (1.25, 1.67); female rate ratio: 1.41 (1.14, 1.75)), and greater STI transmission knowledge (male rate ratio: 1.28 (1.07, 0.54); female rate ratio: 1.41 (1.06, 1.88)) [54]. Okonofua et al. (2003) evaluated an intervention to improve STI treatment seeking that incorporated education on STI prevention and referrals to STI services in a school-based curriculum as well as series of community educational lectures and found that youth who received the intervention reported significantly increases in STI knowledge (mean increase in STI’s named was .63 compared with the control group, 95% CI: 0.39–0.86) and STI treatment-seeking (from 17.5 to 40.7%; OR = 3.24, 95% CI: 1.84–5.74) [30]. The authors also reported significantly reduced STI prevalence (OR = 0.68,95% CI = 0.48–0.95) compared with youth in control schools at post-intervention. In the school-based HIV and IPV prevention program reported by Matthews et al. (2016), youth who received the intervention reported significantly greater HIV knowledge than those in the control arm at 12-month follow-up (*p* < .01) [69]. Finally, the HIV/STI prevention intervention for youth in Vietnam also reported significantly greater HIV/STI knowledge among youth who received the intervention (*p* < .05) compared with youth in the control condition at post-intervention and 6-month follow-up [63]. Jones et al. (2013) also found increased HIV knowledge at post-intervention among young couples completing the HIV-risk reduction intervention (F(1, 476) = 13.9, *p* < .001) [70]. All studies were randomized controlled trials.

### Sexual self-efficacy

Four studies reported significant effects for sexual self-efficacy, including condom negotiation self-efficacy, self-efficacy for safe sex, and sexual refusal self-efficacy. In the study of an HIV prevention intervention among youth in Zimbabwe mentioned above, Cowan et al. (2010) found that female youth who participated in the intervention reported significant increases in condom negotiation self-efficacy compared with those in the control condition (AOR:1.17; 95% CI: 0.95–1.43), but no significant differences were found for male youth [25]. Similarly, Baioochi et al. (2017) investigated a school-based program to increase girls’ empowerment and boys’ education about gender equity among youth (ages 10–16) in Kenya, and those who received the intervention reported significant increases in general self-efficacy, described as perceived ability to cope with stress and manage difficulties (mean score increase = .19, 95% CI: 0.08–0.39) [46]. Finally, in a study on a church-based HIV prevention intervention for youth and families in Kenya, youth who received the intervention reported increases in self-efficacy for safe sex compared with controls at 1-month follow-up (b = .41, *p* < .01), but effects were not sustained at 3-month follow-up [47]. In the study on the HIV prevention intervention among youth in Vietnam, youth who received the intervention reported increases in condom use self-efficacy and condom negotiation self-efficacy compared with those in the control condition (*p* < .001) at post-intervention and 6-months follow-up. All studies were randomized controlled trials [63].

### SRH knowledge

We identified three studies that found significant intervention effects for improving accurate SRH knowledge. Leventhal et al. (2016) examined a psychosocial curriculum plus a health curriculum compared with the psychosocial curriculum alone and a control condition delivered in schools among girls in India (average age = 13) [64]. The authors found that those who received both the health and psychosocial curriculum combined and the health curriculum alone reported significantly greater reproductive health knowledge (i.e., menstrual hygiene) than girls in the control condition (*p* < .05) at post-intervention. The study by Ross et al. (2007) among youth in Tanzania reported that those who received the SRH intervention reported significant increases in pregnancy prevention knowledge (male rate ratio: 1.66; 95% CI: 1.55–1.78; female rate ratio: 1.58; 95% CI: 1.26–1.99) compared with those in the control condition [54]. In the Mercy Corps (2015) study evaluating Safe Spaces alone and Safe Spaces plus a livelihood-training component among girls in Niger, both groups demonstrated significantly greater SRH knowledge compared with controls [31]. Girls in Safe Spaces were 69.7% more likely to know one benefit of delaying pregnancy to 18 or older (*p* < .01), whereas girls in the livelihood program were 27.7% more likely (p < .05).

### Gender-based violence and gender equity

Five studies were identified that reported improvements in gender-based violence. Matthews et al. (2016) found that youth who received the HIV and intimate partner violence (IPV) prevention intervention were significantly less likely to report experiencing sexual violence than those in the control arm at 12-month follow-up (35.1 vs. 40.9%; OR 0.77, 95% CI 0.61–0.99; t [30] = 2.14) [69]. Devries et al. (2017) investigated the Good Schools Toolkit, which includes education on sexual and emotional violence and relationship power, among youth in Uganda (ages 11–14) [58]. The authors found that those who received the intervention reported significant reductions in levels of violence (male student AOR = 0.34, 95% CI: 0.22–0.53; female students AOR = 0.55, 95% CI: 0.36–0.84). In the school-based curriculum developed by Baiocchi et al., (2017), results also showed that following the girls’ empowerment and boys’ gender equality education intervention components, there was an estimated 3.7% decrease (*p* = 0.03; 95% CI: 0.4–8.0), risk of sexual assault in the intervention group [25]. Raj et al. (2016) found that of those participating in the gender equity intervention, women were less likely to report IPV (AOR = 0.48, *p* = 0.01), and men were less likely to report acceptance of IPV in comparison to those in the control condition at 18-month follow-up (AOR = 0.51, *p* = 0.004) [66]. In the couples-based HIV-risk reduction intervention in South Africa, there was a significant reduction in reported acts of violence and verbal aggression among those in the intervention group (McNemar’s test, *p* = 0.001 for intervention, *p* = 0.49 for control; McNemar’s test, p = 0.01 for intervention, *p* = 0.10 for control, respectively) [70].

### SRH intervention components

The SRH components of intervention packages demonstrating significant effects on SRH outcomes focused primarily on education/knowledge building and skills building. The most frequent educational components were: SRH education (i.e., puberty, reproductive anatomy, pregnancy; 7/17), HIV/STI prevention education (7/17), gender equity education (6/17), and education about effective contraceptive methods (including condom use) (6/17). Education on gender-based violence was the next most common component (4/17). Three studies also incorporated parent education sessions on youth SRH, and three included education on healthy relationships. For skill-building, sexual decision making skills components were incorporated in three studies, and building condom use skills through demonstration and practice was incorporated into those interventions focused on improving knowledge of effective contraceptive methods. Three studies incorporated youth friendly services as an intervention component to increase youth access to SRH services. For studies reporting pregnancy prevention as an SRH intervention component, we coded pregnancy prevention as SRH education.

### Psychosocial intervention components

Of the 17 studies that reported significant intervention effects on SRH outcomes, approximately 50% (9/17) incorporated psychosocial components into the intervention package. The most common psychosocial components included were assertiveness training (4/17) communication skills (4/17), and problem solving (4/17), (see Fig. 3). Other common psychosocial intervention components included emotion regulation, goal setting, resilience-building, cognitive, modeling, coping skills, and psychoeducation-staff.

### Intervention delivery information

The majority of the effective SRH interventions were delivered in school settings (13/17). Three of these interventions were delivered in multiple settings that included schools and either community centers or clinics. Other intervention delivery settings were clinics (3/17), churches (1/17) or village community settings (1/17). The most frequent delivery format for interventions was group-based (15/17), with two group-based interventions delivered to female youth only. Four interventions involved an individual counseling component, four involved parent education and discussion groups, and four involved a community component (i.e., community outreach activities, public lectures, etc.). Multi-component intervention approaches that included youth groups and either a parent group or community component (or both) were also reported in 8/17 studies. Three studies incorporated peer outreach training. Two studies involved couples-based interventions that included male partner involvement. Four studies incorporated service provider training for SRH services, and three of these implemented youth friendly health services as an intervention component. Only four studies described both facilitator training and supervision procedures; five studies provided some description of facilitator training procedures but failed to report on supervision. Interventions varied greatly in terms of session frequency, length, and overall duration of the intervention (see Table 1).

### Methodological quality

**Fig. 4:**
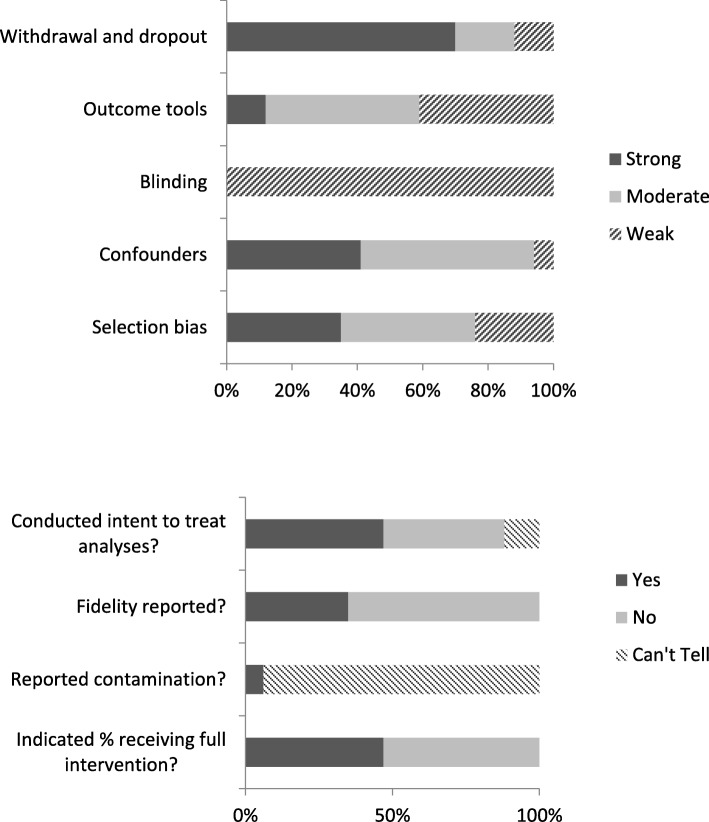
Quality assessment results (n = 17) with modifications to EPHPP criteria. Blinding criteria modified: a rating of strong was assigned when both assessors and participants were blinded, a rating of moderate was assigned when either assessors or participants were blinded, and a rating of weak was assigned when neither were blinded or it could not be determined. Confounders criteria modified: age, gender and SES were considered as the important potential confounders

Results of the quality assessment rating in accordance with the EPHPP criteria are displayed in Fig. 4. Studies reported strong participant study completion rates, with 11 studies indicating completion rates of 80% or higher, and only two studies failed to report participant withdrawal and dropout information. There were also several methodological weaknesses of these studies. A major methodological weakness was the failure to blind both participants and assessors or the lack of reporting about whether blinding occurred. Only one study reported that assessors were blinded, and no studies reported that participants were blinded to study condition. Another weakness was reporting of whether contamination likely occurred; only one study reported that participants in the comparison condition likely had some exposure to the intervention program. Many studies also failed to report information on the reliability or validity of assessment tools, with only four studies including information on validity and eight on reliability. Finally, only six studies described processes through which fidelity to the intervention was assessed or monitored, and no studies reported results of fidelity assessments.

## Discussion

The current systematic review aimed to fill gaps in existing research by focusing on more rigorously evaluated SRH interventions for young people in LMIC and humanitarian settings to better understand which types of interventions demonstrated effectiveness. The review also aimed to examine the inclusion of psychosocial intervention components in those SRH interventions identified as effective. Findings suggest that there is preliminary support for the effectiveness of several evidence-based SRH interventions for young people in humanitarian and low resource settings, but additional research is needed, including replication studies, studies with longer follow-up periods, and studies focused on feasibility, implementation and sustainability of interventions on a broader scale. This is consistent with conclusions of the few prior reviews on SRH services for adult populations during humanitarian crises—the evidence-base for effective interventions is limited and more implementation research is needed [7, 8, 83].

Of those SRH interventions determined to be effective, the most common SRH outcomes that improved over time were (a) effective contraception and condom use skills and (b) HIV and STI prevention knowledge) [30, 31, 48, 54, 59, 62, 63, 66, 68–70, 74]. Findings suggest that effective evidence-based approaches to improve knowledge about effective contraception and prevention of HIV and other STIs among young women and men in humanitarian and LMIC settings are available, but must be adapted to context and the realities of humanitarian emergencies (such as high levels of trauma exposure and loss). For resource limited areas and fragile contexts in which implementing high intensity interventions are challenging, incorporating education about these two key components of SRH in a trauma-informed manner could be viable options. Lower intensity approaches might also be more easily integrated into existing service systems or school-based health curriculum and still yield important improvements in SRH. Given that many of the effective interventions consisted of lengthy periods of delivery (e.g., 1–2 years of a program) that involved a high level of coordination and training, identifying key content areas of effective SRH interventions is crucial for identifying potential “active ingredients” to include in a deployment-focused model [84] to facilitate greater feasibility and adoption of integrated SRH and psychosocial interventions for young people in the sorts of delivery settings available for populations affected by humanitarian emergencies. While beyond the scope of the current review, future research might explore whether the methods outlined in the common elements approach could be validly applied to identify common SRH elements of those interventions that failed to demonstrate effectiveness, which could help to better disentangle which components are less effective across studies.

Effective interventions tended to be delivered in a group format with similar age peers. The majority of these group-based interventions for school-aged youth were delivered in schools either during regular hours or as an extracurricular activity after school. There were vast differences in delivery format for school-based interventions, with intervention length ranging from six sessions to 24 sessions and session duration ranging from 40 min to 120 min; therefore, it is difficult to ascertain which characteristics of SRH intervention delivery in school settings might be optimal for improving SRH outcomes or engaging young people. Multi-component interventions for adolescents that involved parents and community members were also common among those SRH interventions demonstrating effectiveness [25, 47, 62, 63, 74]. This suggests that engaging parents and key community members in talks, support groups, and educational sessions related to SRH among youth could be an important component of effective SRH interventions. Engaging parents and community leaders could help raise awareness about youth sexuality and SRH concerns as well as decrease stigma related to seeking SRH services among youth. Interventions that involved parent sessions also focused on improving communication between young people and their families around SHR topics, which suggests that this may also be an important aspect of SRH interventions targeting youth.

Of those interventions that showed improvements in gender-based violence or gender equity awareness, nearly all interventions involved both young men and women, though interventions varied in terms of whether groups were separated by sex/gender and whether they were couples-based (*n* = 2 couples interventions). For the two couples-based interventions, one consisted of four weekly 90–120 min couples’ sessions, whereas the other consisted of two individual sessions delivered by males to males and one couples-based session. This highlights the potential importance of involving and engaging young men for improving young women’s sexual health, empowerment, and well-being. Investment in interventions that engage men in reproductive health, maternal health, and gender-based violence in LMICs has increased considerably in recent years [85–87]. thus further supporting the notion that male engagement is a critical component of addressing SRH in LMICs and humanitarian emergencies. Implementing SRH interventions that involve young men may be particularly important during adolescence and young adulthood, as this is a developmental period when norms and beliefs about gender are shaped and solidified.

Nearly half of the SHR interventions designated as effective incorporated psychosocial components into their session content [25, 46, 47, 59, 63, 64, 69, 70]. However in those instances when psychosocial outcomes were also measured, information was not consistently provided as to whether participants reported improvements in psychosocial or mental health outcomes. Additional, potential associations between improvements in mental health or other psychosocial outcomes and SRH outcomes were not reported. Given the high prevalence of mental health issues among refugee and displaced populations of youth as well as the higher prevalence among women in comparison to men [18, 20]. Future research would benefit from examining the links between psychosocial and SRH outcomes in intervention packages that include both components and to what degree the backdrop of trauma, common in populations affected by humanitarian emergencies, requires additional attention. In particular, assertiveness training and communication skills could be important psychosocial intervention components that could be incorporated into trauma-informed intervention models to promote SRH outcomes like sexual self-efficacy (both condom/contraceptive negotiation self-efficacy and sexual refusal self-efficacy) and sexual decision-making skills. Additional research is needed to better understand which common psychosocial practice elements might be linked to improved SRH outcomes in LMIC and humanitarian contexts. As with SRH components, future research might consider whether the common practice elements approach could be feasibly applied to identify common psychosocial components of ineffective interventions, which could potentially provide information on which components are less useful or pertinent in achieving intervention benefits.

### Limitations

We restricted our search to those studies published in the English language, which may have limited the number of articles retrieved from our search. Qualitative studies were excluded from the search, which can often provide rich information regarding feasibility and acceptability of interventions as well as guide selection of culturally appropriate incentives to improve participation and retention during intervention implementation. We limited our search to randomized controlled trials (with the exception that quasi-experimental studies conducted in humanitarian settings or the Middle East were included) in order to gather information on those SRH interventions that were evaluated through rigorous scientific methods, which may have limited the studies retrieved in low resource settings that lack funding and expertise for more costly evaluations of interventions. Because we restricted our participant age criteria to exclude studies that included participants ages 10 and under, we may have excluded some effective SRH interventions for adolescent populations if the study included younger participants. It should be noted that our criteria for including an intervention as effective were that the intervention demonstrated significant improvements in one or more of the reported SRH outcomes over time. There were several instances in which an intervention showed significant effects on one SRH outcome, while failing to impact several others (or impacting outcomes in unexpected directions for particular subgroups). We retained these studies because they still met our inclusion criteria, but the inconsistency in improvement across SRH outcomes raises some questions as to thus overall effectiveness of those interventions. Inclusion of some studies conducted in upper-middle income countries could be less relevant for populations in the Middle East and humanitarian settings due to cultural, social, economic, and political differences among these contexts; thus their findings may not necessarily generalize to humanitarian contexts. Heterogeneity of measurement tools used to assess SRH outcomes across studies and lack of psychometric information on outcome tools obfuscated comparison of effects across studies.

## Conclusion

The current review offers a comprehensive summary of novel and effective SRH interventions for young people in LMIC and humanitarian settings. It describes these SRH interventions, highlights their novel strategies where possible, and describes the range of SRH outcomes that showed significant improvements. The review also includes a thematic analysis of intervention delivery characteristics, core intervention components and provides a quality assessment of the studies reporting effective SHR interventions for young people. Findings of this review provide some potentially useful insights for adaptation of evidence-based interventions for young people in different contexts and could inform decisions of key stakeholders to further invest in particular interventions for broader dissemination and scale-up in LMICs, the Middle East and humanitarian settings. Findings of this review may also be useful for public health and policy workers, as it could broaden their understanding of what works more effectively and for whom in fragile contexts, with the aim of improving SRH outcomes and related SRH service use among young people in these settings.

## Data Availability

The datasets used and/or analyzed during the current study are available from the corresponding author upon reasonable request.

## Abbreviations

SRH: Sexual and Reproductive Health
LMICs: Lower and Middle Income Countries
STIs: Sexually Transmitted Infections
HIV: Human Immunodeficiency Virus
MISP: Minimal Initial Service Package for Reproductive Health in Crisis
PRISMA: Preferred Reporting Items for Systematic Reviews and Meta-analysis
EPPHP: Effective Public Health Practice Project
IPV: Intimate Partner Violence
WHO: World Health Organization
PMTCT: Prevention of Mother-to-Child Transmission
